# Easy and Affordable: A New Method for the Studying of Bacterial Biofilm Formation

**DOI:** 10.3390/cells11244119

**Published:** 2022-12-19

**Authors:** Dan Alexandru Toc, Alexandra Csapai, Florin Popa, Catalin Popa, Violeta Pascalau, Nicoleta Tosa, Alexandru Botan, Razvan Marian Mihaila, Carmen Anca Costache, Ioana Alina Colosi, Lia Monica Junie

**Affiliations:** 1 Department of Microbiology, Iuliu Hatieganu University of Medicine and Pharmacy, 8 Victor Babeș Street, 400000 Cluj-Napoca, Romania; 2 Materials Engineering Department, Technical University of Cluj-Napoca, 103-105 Muncii Ave., 400641 Cluj-Napoca, Romania; 3 Molecular and Biomolecular Physics Department, National Institute for Research and Development of Isotopic and Molecular Technologies, 67-103 Donat Street, 400293 Cluj-Napoca, Romania; 4 Faculty of Medicine, Iuliu Hatieganu University of Medicine and Pharmacy, 8 Victor Babeș Street, 400000 Cluj-Napoca, Romania; 5 Department of Ophthalmology, Centre Hospitalier Régional d’Orléans, 14 Av. de l’Hôpital, 45100 Orléans, France

**Keywords:** biofilm, dynamic study, biofilm production, enterococcus faecalis, staphylococcus aureus, klebsiella pneumoniae, pseudomonas aeruginosa

## Abstract

Background: Bacterial biofilm formation (BBF) proves itself to be in the spotlight of microbiology research due to the wide variety of infections that it can be associated with, the involvement in food spoilage, industrial biofouling and perhaps sewage treatment. However, BBF remains difficult to study due to the lack of standardization of the existing methods and the expensive equipment needed. We aim to describe a new inexpensive and easy to reproduce protocol for a 3D-printed microfluidic device that can be used to study BBF in a dynamic manner. Methods: We used the SolidWorks 3D CAD Software (EducationEdition 2019–2020, Dassault Systèmes, Vélizy-Villacoublay, France) to design the device and the Creality3D Ender 5 printer (Shenzhen Creality 3D Technology Co., Ltd., Shenzhen, China) for its manufacture. We cultivated strains of *Enterococcus faecalis, Staphylococcus aureus, Klebsiella pneumoniae and Pseudomonas aeruginosa*. For the biofilm evaluation we used optical coherence tomography (OCT), scanning electron microscopy (SEM), Fourier Transform Infrared (FTIR) spectroscopy and crystal violet staining technique. Results: Based on the analysis, *Enterococcus faecalis* seems to produce more biofilm in the first hours while *Pseudomonas aeruginosa* started to take the lead on biofilm production after 24 h. Conclusions: With an estimated cost around €0.1285 for one microfluidic device, a relatively inexpensive and easy alternative for the study of BBF was developed.

## 1. Introduction

Bacterial biofilm formation (BBF) is usually a multi-step process, involving the attachment of the bacteria to the substrate, the formation of microcolonies, the growth and maturation of the microcolonies into the mature biofilm and finally the dispersion of the mature biofilm [1,2,3]. In recent years, new data have shown the frequent involvement of biofilms in human pathologies. Examples of biofilm mediated infections include endocarditis, prosthetic device infections, and catheter-related urinary tract infections and bacteremia [1,4,5,6,7,8]. Unsurprisingly, there is a brand-new worldwide trend to study BBF, its involvement in human infections and the means of eradication. Existing data shows how difficult the eradication of a mature biofilm from different substrates can be, and with the emerging threat of antimicrobial resistance there is a chance that biofilm eradication will play a pivotal role in the future of healthcare [9,10]. Besides infections, biofilms are involved in other relevant biological functions and anthropic situations, such as food spoilage, that makes them a constant presence in the human world. It is also important to acknowledge their involvement in industrial biofouling and future possibilities for their usage in sewage treatment [11,12,13].

There are many ways to study BBF, each with their own advantages and disadvantages. BBF study can be easily organized in a static and a dynamic way. Some of the most commonly used ways to study BBF are in a static way, with the microtiter plate technique being in the spotlight [14]. This technique has certain limitations, such as poor reproducibility, exhaustion of nutrients and difficult direct inspection. However, it can be successfully used for the screening of biofilm formation capacity since it is inexpensive and does not need specific equipment [14]. Regarding the dynamic study of biofilm formation, there are many devices available that can provide a wide variety of information such as the Calgary device, the Robbins device, the Drip Flow Biofilm Reactor or Flow Chamber [15,16,17,18]. One of the common inconveniences of these devices is the price, since most of them are relatively expensive [14]. There is no doubt that there is an acute need for deeper understanding of BBF in a dynamic way in order to properly tackle the future challenges of this research niche. Dynamic study of biofilm formation has the potential to better describe the stages of biofilm development and its dynamics. By using microfluidic devices, we might be able to accurately describe the interaction of BBF and blood vessels or some synthetic structures and thus imitate the human body environment [19].

A 3D printed device using inexpensive starting materials may serve as a simple alternative to conventional methods for BBF studies. There have been several attempts already to create such a device; however, the lack of uniformity in the data they produce makes this approach still questionable [14].

Various manufacturing methods such as wet etching, reactive ion etching, conventional machining, photolithography, soft lithography, hot embossing, injection molding, laser ablation, in situ construction, and plasma etching have been previously used for the fabrication of microfluidic devices [20,21,22,23]. The presented design has a major advantage in being H-type structured, the double input making it possible to cultivate polymicrobial biofilms and to test for the antibiofilm effect of different substances. This offers a great versatility in the type of research protocols it can be used for.

The aim of this study is to describe the BBF in a custom-made 3D printed microfluidic device and to provide a step-by-step guide to printing, assembling and biofilm cultivation as means to describe the biofilm growth.

## 2. Materials and Methods

### 2.1. 3D-Printing of the Devices

**Figure 1:**
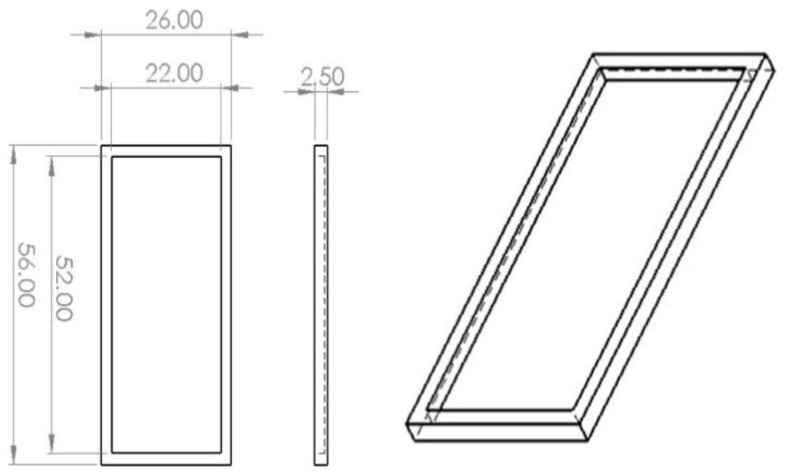
General characteristics of the 3D-printed plate device (in mm).

**Figure 2:**
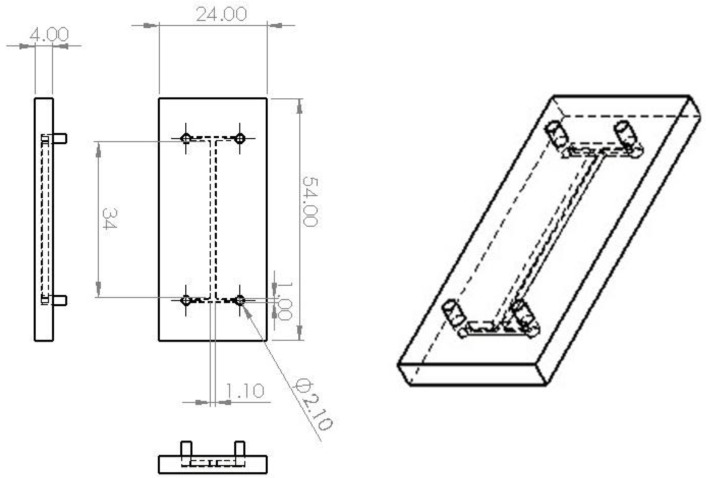
General characteristics of the 3D-printed microfluidic device (in mm).

Two device types were printed—one plate type device for static BBF testing (Figure 1) and one microfluidic device for dynamic BBF testing (Figure 2). In order to properly describe the characteristics of BBF inside the device, we initially grew, analyzed and quantified BBF on the 3D-printed plate so that the interaction of bacteria with the polylactic acid (PLA) surface becomes clear. We chose a relatively simple design for the plates since our main interest was to observe the way bacteria interact with the surface and how much biofilm they produce.

We have developed an “H” type microfluidic device for drug delivery, cell selection and dynamic study of biofilm growth using additive manufacturing (AM) as a manufacturing method. The system was designed using the SolidWorks 3D CAD Software (EducationEdition 2019–2020, Dassault Systèmes, Vélizy-Villacoublay, France), having the overall dimensions of 54 mm × 24 mm × 4 mm (L × W × H). The microfluidic device was printed using the Creality3D Ender 5 printer (Shenzhen Creality 3D Technology Co., Ltd., Shenzhen, China) together with a 1.75 mm PLA filament (VerbatimTM—Mitsubishi Kagaku Media Co., Ltd., Tokyo, Japan). The structure of the device includes two 1 mm inlet channels, two 2 mm outlet channels and one 2 mm wide, 34 mm long central channel. The height of the channels is 2.5 mm. Using the BuildFX PRO DC and a UV lamp (Dymax light curing Systems, BlueWave™ 50), the inlet/outlet adapters were soldered to the device, providing a complete sealing of the microsystem. The pumping process of the fluids within the channels was done using an SP230iwZ Syringe pump (WPI) (Figure 2).

### 2.2. Bacterial Cultivation and Biofilm Formation

Since biofilm formation is a characteristic of many bacteria, it was challenging choosing the most relevant bacterial species. For the initial evaluation, we settled for some of the most common bacteria involved in biofilm-related infections: two Gram positive bacterial species *Staphylococcus aureus* ATCC 25923 and *Enterococcus faecalis* ATCC 29212; and two Gram negative bacterial species *Klebsiella pneumoniae* ATCC 13883 and *Pseudomonas aeruginosa* ATCC 27853.

To properly evaluate the utility, feasibility and reproducibility of the protocol and the technique described, the experiments were reproduced using 5 different clinical isolates of each bacterial species of choice (*Enterococcus faecalis, Staphylococcus aureus, Klebsiella pneumoniae* and *Pseudomonas aeruginosa)*, from the Iuliu Hatieganu University of Medicine and Pharmacy Cluj-Napoca, Department of Microbiology collection. Each bacterial strain evaluated was previously identified using the Vitek^®^ 2 Compact (bioMerieux, Marcy—l’ Étoile, France).

For preparation of bacteria for biofilm cultivation, each species was cultivated on Columbia agar + 5% sheep blood (bioMerieux, Marcy—l’ Étoile, France) for 24 h at 37 °C. Each culture was then standardized by suspension of colonies into saline solution until a MacFarland Standard of 3 was achieved. This standard corresponded to 9 × 10^8^ CFU/mL.

For biofilm static cultivation in 3D-printed plates, 5 mL bacterial solution and 5 mL Mueller Hinton broth (MBH, Bio-Rad, Marnes-la-Coquette, France) were used. The plates were cultivated at 37 °C for 6 h, 12 h, 24 h, 3 days, 5 days and 7 days.

**Figure 3:**
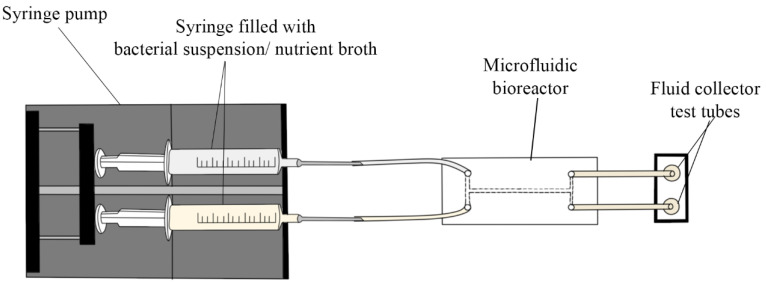
The 3D printed microfluidic bioreactor.

For biofilm cultivation in the 3D-printed microfluidic bioreactor, the same quantities as previously described were used, but the bacterial solution and the broth were drawn in two syringes (5 mL each) connected to the silicon tubes attached to the inlets of the system, as presented in Figure 3. The flow rate used was of 0.3 mL/min at room temperature to pump the fluid through the microsystems. Once the flow rate stopped, the microfluidic device was cultivated at 37 °C for 6 h, 12 h, 24 h, 3 days, 5 days and 7 days, respectively. This way, the initial step in biofilm formation—adhesion—was analyzed. The 3D-printed microfluidic devices were then sectioned longitudinally, along the main channel, using a Robotec laser cutting machine.

Standardized strains (*Staphylococcus aureus* ATCC 25923, *Enterococcus faecalis* ATCC 29212, *Klebsiella pneumoniae* ATCC 13883 and *Pseudomonas aeruginosa* ATCC 27853) were used in the beginning. Afterwards, the clinical isolates were used to reproduce the experiment. All experiments were performed in triplicate and the results are expressed as the average of the 3 determinations.

### 2.3. Measurements of Developed Biofilms

Bacterial biofilm detection and quantification was determined by several methods including a modified crystal violet technique, optical coherence tomography (OCT), scanning electron microscopy (SEM) and Fourier Transform Infrared (FTIR) Spectroscopy.

#### 2.3.1. Modified Crystal Violet Technique

**Figure 4:**
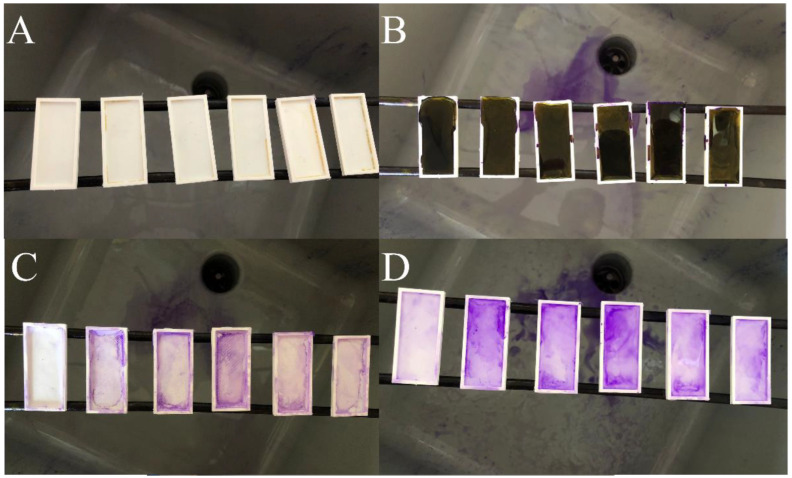
Modified crystal violet staining for quantitative assessment of BBF on the 3D-printed plates. ((**A**) = initial aspect of the BBF after incubation. (**B**) = crystal violet staining. (**C**) = aspect of the BBF after washing the stain with water. (**D**) = discoloration using alcohol-acetone.).

The determination of total biofilm quantity was performed using the crystal violet technique previously described for a similar setting [24]. For the 3D printed plates, a sequence of staining with crystal violet 1% for 2 min was used: three consecutive rounds of washing-discoloration using alcohol. The final solution was stored in a glass tube and absorbance determined using a UV-VIS spectrometer at 590 nm (Figure 4). The same sequence was used for the 3D printed microfluidic device; however, the substances were pushed through the microfluidic device with the syringe pump.

#### 2.3.2. Optical Coherence Tomography (OCT), Scanning Electron Microscopy (SEM) and Fourier Transform Infrared (FTIR) Spectroscopy

The proper assessment of the existence, the quality and topography of the BFF used the following imaging techniques: OCT, SEM and FTIR. The OCT measurements were performed by an OCTG-1300 Handheld Scanner (THORLABS GmbH, Luebeck, Germany) equipped with an OCTH-LK30 lens kit (THORLABS GmbH, Luebeck, Germany). The images were further processed using Adobe Photoshop to emphasize the biofilm by coloring in a different shade for each strain.

The SEM images were obtained using a JEOL/JSM 5600-LV (JEOL Ltd., Tokyo, Japan). For this technique samples were washed with absolute ethanol and were further carbon layered with plasma sputtering equipment. 

In order to record the FTIR spectra of the bacterial biofilms formed on the surface of the PLA printed plates, a Spectrum BX FTIR spectrometer (PerkinElmer, Sunnyvale, CA, USA) provided with an Attenuated Total Reflectance (ATR PIKE MIRacleTM, Madison, WI, USA) with a diamond crystal plate was used. The resolution was 4 cm^−1^ in the range of 4000–800 cm^−1^. For this characterization step, no sample preparation was necessary. Using the attenuated total reflection (ATR) accessory alongside the traditional infrared spectroscopy allowed for a direct visualization of the characteristic spectra of the biofilms.

### 2.4. Statistical Analysis

For the analytical statistics of the collected data, we used SPSS Statistics version 25 (IBM, New York, NY, USA). We measured the biofilm concentration (expressed in mol/L) and we collected the data in a spreadsheet. Given that biofilm concentration represents a continuous variable, we tested the distribution of the data using the Shapiro–Wilk test in order to categorize it as either normal or non-normal, and we applied the relevant statistical tests (parametric tests—either *t*-test for comparison between two sets of data or ANOVA for comparison between multiple sets of data—for the former category and non-parametric tests—either Mann–Whitney U test for comparison between two sets of data or Kruskal–Wallis test for comparison between multiple sets of data—for the latter). A *p*-value of less than 0.05 was chosen as proof of statistical significance.

## 3. Results

**Figure 5:**
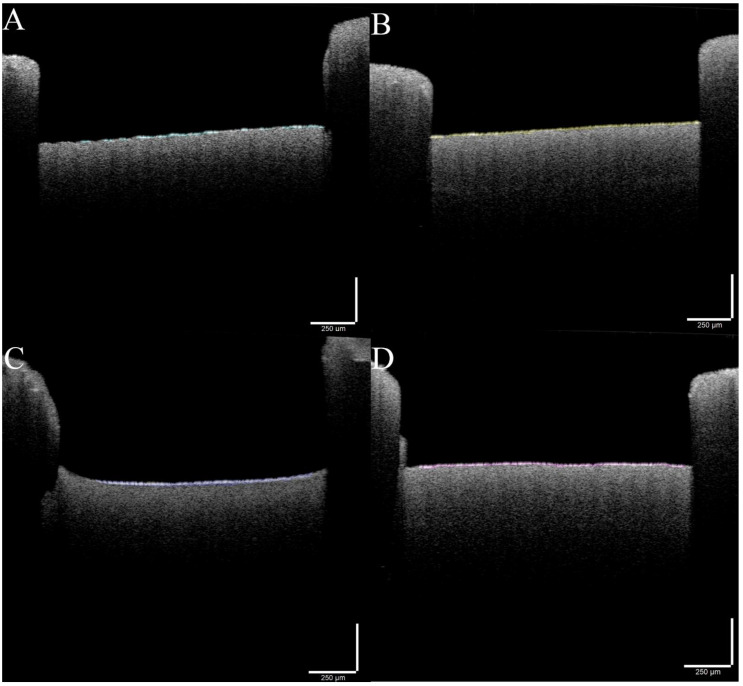
Optical coherence tomography (OCT) images showing the bacterial biofilm. (**A**) = *Enterococcus faecalis* biofilm (green), (**B**) = *Staphylococcus aureus* biofilm (yellow), (**C**) = *Klebsiella pneumoniae* biofilm (blue) and (**D**) = *Pseudomonas aeruginosa* (red). The strains used were *Staphylococcus aureus ATCC 25923*, *Enterococcus faecalis ATCC 29212*, *Klebsiella pneumoniae ATCC 13883* and *Pseudomonas aeruginosa ATCC 27853*.

Based on the previously described protocols, the qualitative assessment of the BBF started with the analysis of the 3D printed surface. In this way, it was proved that BBF can be achieved and quantified by various techniques such as OCT, SEM and FTIR. OCT proved to be quite a revolutionary technique in studying BBF; due to the technology behind it, the samples need to have some specific characteristics that allow the light to pass through the biofilm. Our design along with the laser cutting technique overcame these obstacles and allowed the use of OCT in BBF study. Figure 5 presents the bacterial biofilms on the 3D-printed microfluidic device. We used Photoshop to digitally stain the biofilm structure from the surface, using different colors. This kind of image can be further processed, if needed, to have a deeper understanding of the biofilm architecture.

**Figure 6:**
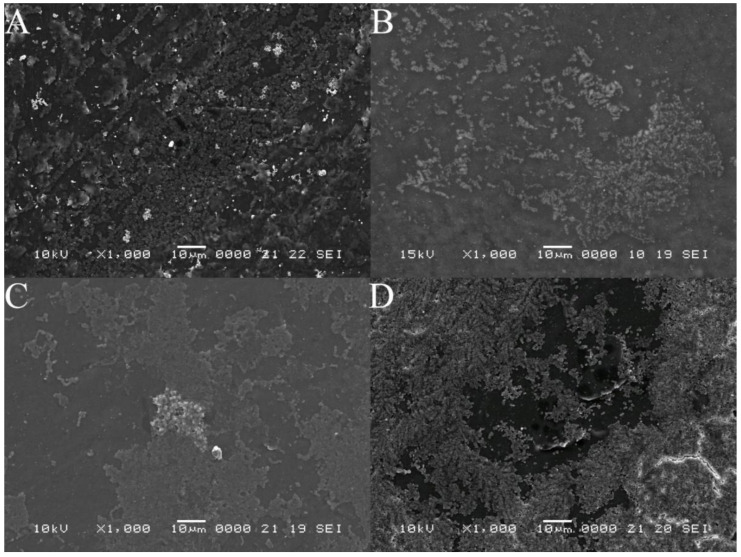
Scanning electron microscopy (SEM) images at ×1000 magnification, showing bacterial growth on the 3D-printed PLA plate. (**A**) = *Enterococcus faecalis*, (**B**) = *Staphylococcus aureus*, (**C**) *= Klebsiella pneumoniae* and (**D**) = *Pseudomonas aeruginosa*. The strains used were *Staphylococcus aureus ATCC 25923, Enterococcus faecalis ATCC 29212, Klebsiella pneumoniae ATCC 13883* and *Pseudomonas aeruginosa ATCC 27853*.

**Figure 7:**
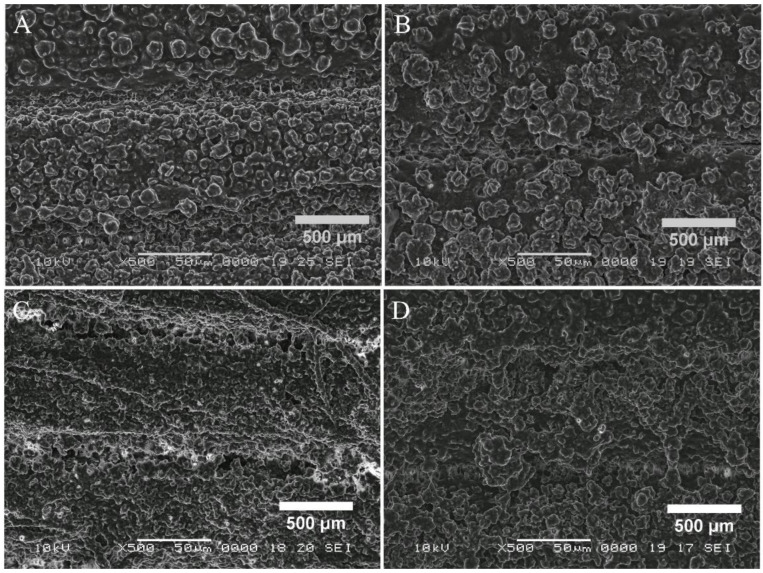
Scanning electron microscopy (SEM) images at ×500 magnification, showing bacterial growth on the 3D-printed PLA microfluidic devices. (**A**) = *Enterococcus faecalis*, (**B**) = *Staphylococcus aureus*, (**C**) = *Klebsiella pneumoniae* and (**D**) = *Pseudomonas aeruginosa*. The strains used were *Staphylococcus aureus ATCC 25923, Enterococcus faecalis ATCC 29212, Klebsiella pneumoniae ATCC 13883* and *Pseudomonas aeruginosa ATCC 27853*.

SEM is also a very useful tool in the BBF study that has been used for quite some time now. Figure 6 and Figure 7 present the BBF on the 3D-printed surface, thus proving that the protocol described is working effectively. The bacterial microcolonies, one of the steps in BBF, can be easily observed.

**Figure 8:**
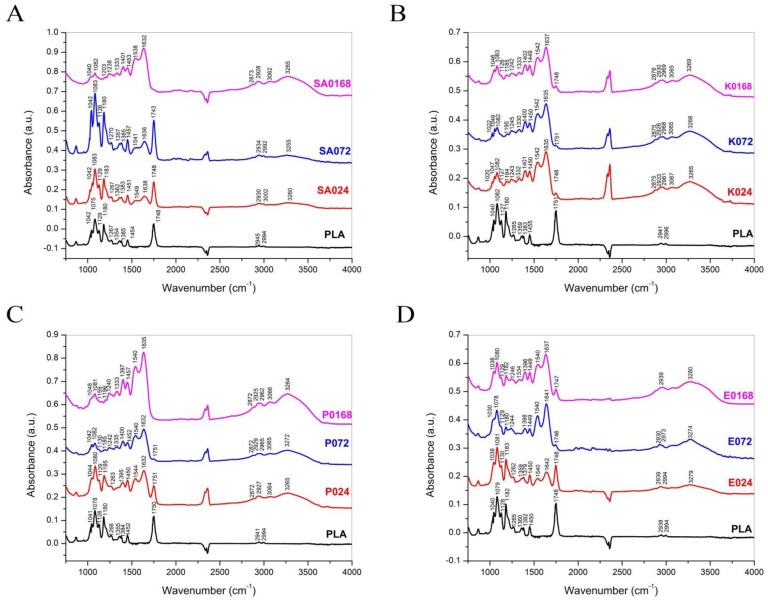
Fourier Transform Infrared (FTIR) Spectroscopy images of biofilm cultivated for 24 h, 72 h and 168 h on the PLA surface. (**A**) = *Staphylococcus aureus* (SA) biofilm at 24 h (SA024), 72 h (SA072) and 168 h (SA0168). (**B**) = *Klebsiella pneumoniae* (K) biofilm at 24 h (K024), 72 h (K72) and 168 h (K0168). (**C**) = *Pseudomonas aeruginosa* (P) biofilm at 24 h (P024), 72 h (P072) and 168 h (P0168). (**D**) = *Enterococcus faecalis* (E) biofilm at 24 h (E024), 72 h (E072) and 168 h (E0168). The strains used were *Staphylococcus aureus ATCC 25923, Enterococcus faecalis ATCC 29212, Klebsiella pneumoniae ATCC 13883* and *Pseudomonas aeruginosa ATCC 27853*.

Fourier Transform Infrared (FTIR) spectroscopy is a relatively new and exceptional tool for the study of BBF. It allows bacterial detection, identification, classification and other quantifications such as cell enumeration, and extracellular polymeric substance characterization which makes this new approach practical in the study of BBF. Figure 8 presents the bacterial biofilm spectra obtained from the 3D-printed plate, using the protocol previously described. The correct identification of the bacteria can be observed based on specific spectra as well as changes in the biofilm polysaccharide and protein components (Amide I and Amide II peaks) as they form in time at 24 h-72 h-168 h on the surface.

Going further, the quantity of bacterial biofilm was assessed using crystal violet technique in both static (3D-printed plates) and dynamic (3D-microfluidic devices) ways. In static conditions, the average concentration of biofilm for *Staphylococcus aureus ATCC 25923* was 779 mol/L at 6 h, 2477 mol/L at 12 h, 341 mol/L at 24 h, 4685 mol/L at 72 h, 5915 mol/L at 120 h and 501 mol/L at 168 h. For *Enterococcus faecalis ATCC 29212* the average concentration of biofilm was 2935 mol/L at 6 h, 4106 mol/L at 12 h, 5982 mol/L at 24 h, 6777 mol/L at 72 h, 5595 mol/L at 120 h and 5303 mol/L at 168 h. *For Pseudomonas aeruginosa ATCC 27853* the average concentration of biofilm was 2585 mol/L at 6 h, 3567 mol/L at 12 h, 3956 mol/L at 24 h, 7858 mol/L at 72 h, 6695 mol/L at 120 h and 6015 mol/L at 168 h. For *Klebsiella pneumoniae ATCC 13883* the average concentration of biofilm was 245 mol/L at 6 h, 2785 mol/L at 12 h, 2756 mol/L at 24 h, 2886 mol/L at 72 h, 2673 mol/L at 120 h and 2287 mol/L at 168 h.

The static and dynamic measurements of the bacterial biofilm produced by the wild strains (5 of each species) as well as a statistical comparison between the two techniques are presented in Table 1.

Furthermore, a comparison of the bacterial biofilm concentration across the tested species at each of the six time periods in both static and dynamic conditions was performed.

**Figure 9:**
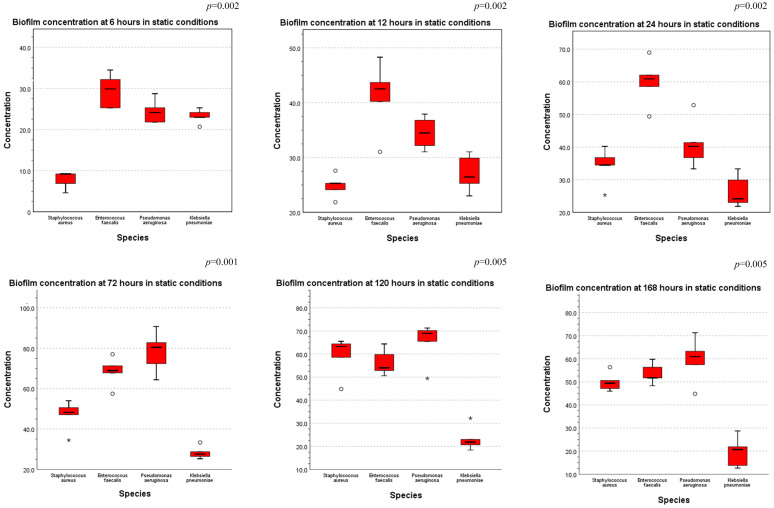
Comparison of biofilm concentration across all species tested in static conditions, at 6 h-12 h-24 h-72 h-120 h-168 h (ANOVA test performed—*p*-values displayed).

In static conditions, a statistically significant difference was observed between the investigated species, as seen in Figure 9, with *Enterococcus faecalis* producing the most biofilm quantity at 6 h-12 h-24 h and *Pseudomonas aeruginosa* at 72 h-120 h-168 h.

**Figure 10:**
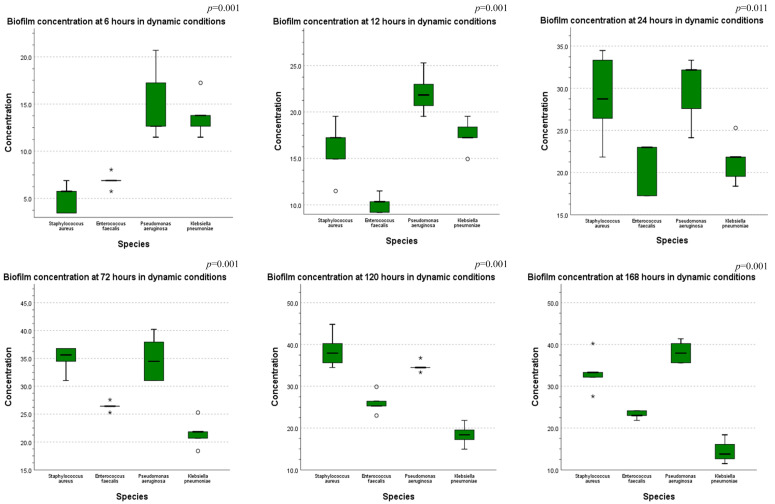
Comparison of biofilm concentration across all species tested in dynamic conditions, at 6 h-12 h-24 h-72 h-120 h-168 h (ANOVA test performed—*p*-values displayed).

Figure 10 presents the results from the comparison of biofilm production in dynamic conditions, showing a statistically significant difference between the investigated species, with *Pseudomonas aeruginosa* producing the most biofilm at almost all the time stamps.

## 4. Discussion

This paper provides a comprehensive study of the potential use of 3D printed PLA surfaces for the growth and analysis of bacterial biofilms in static and dynamic ways. For describing the biofilm, we used some several methods such as FTIR, OCT, SEM and crystal violet staining, proving the feasibility of the protocol described.

A reliable and relatively inexpensive tool for the characterization of the biofilms formed on the 3D printed PLA surfaces is Fourier transform infrared (FTIR) spectroscopy [25,26]. In this study, ATR-FTIR spectroscopy was used for the detection of the specific bacterial biofilms on the printed surfaces. As can be observed in the presented spectra (Figure 7), for each sample, similar intensity peaks are noticeable: at around 1750 and 1080 cm^−1^ the peaks correspond to the carbonyl and C–O–C stretching, with the peaks at 1750 cm^−1^ strongly attenuated for the samples incubated for 168 h; at about 1450 cm^−1^ another peak represents the C–H stretching in methyl groups, with peaks between the values of 1454–1456 (slightly shifted) corresponding to CH3 symmetric deformation vibration; peaks at 1126–1127 cm^−1^ are attributed to the CH3 rocking vibration, and at 1042–1046, peaks are assigned to C-CH3 stretching [27,28]. These peaks are indicative of the PLA substrate present in each of the prepared samples.

Biofilm characteristic absorption peaks are noticeable in the regions attributable to lipids (3000–2800 cm^−1^), with peaks at 2872, 2873, 2875 and 2876 cm^−1^, representing the typical C–H stretching vibrations (nC–H) corresponding to the CH3 and >CH2 functional groups, present in fatty acids and lipids, to proteins (1705–1600 cm^−1^) with peaks around 1632–1638 cm^−1^ attributed to amide I bands, > C = O stretching and C–N bending of protein and peptides amide, at about 1540–1548 cm^−1^ representing the amide II, N–H bending, C–N stretching of proteins and peptides, and to polysaccharides (1200–950 cm^−1^) [29,30,31]. These peaks can be associated with the structure of outer membrane, extracellular matrix, and the bacterial cell wall.

SEM images were used for the investigation of the surface topography of the bacterial biofilms formed both on the plates and inside the microfluidic devices. As can be observed, for the biofilms grown on the plates, the cellular structure is more defined, with recognizable individual cells, regardless of the bacterial strain. The lack of visible deposition lines suggests the development of a thicker biofilm layer, or several layers deposited on the printed plates, a structure favored by the experimental parameters. The SEM images for the biofilm formed inside the microfluidic devices (Figure 7) presents more disorganized structures, with defined colonies, but no identifiable singular cells. This phenomenon could be explained by the experimental conditions, such as the sheer stress and flow rate present inside the microfluidic channels [24]. Another noticeable aspect is the arrangement of the colonies along the deposition lines for the *Enterococcus* and *Klebsiella* biofilms, with cervices between the filaments, and small connection areas between colonies. The *Staphylococcus* and *Pseudomonas* biofilms appear more uniformly distributed along the filament deposition lines.

As can be observed in the OCT images (Figure 5), the deposition of biofilm inside the microfluidic devices is not uniform along the length of the channel, due to process parameters such as sheer stress and flow rate, which can lead to an irregular detachment of the biofilm. The thickness of the biofilm can vary both depending on the type of bacteria and its capacity to form biofilms, as well as on the investigated area (with thicker biofilm layers visible closer to the entrance and towards the center of the channels, and thinner layers towards the end of the channel [24].

Quantitative assessment of the biofilm produced by the wild strains analyzed showed several similarities with the results from other studies which emphasize the accuracy of the results and confirm the utility of this working protocol. However, comparing the Gram-positive bacteria from our study, *Enterococcus faecalis* and *Staphylococcus aureus*, we noticed an increased biofilm production *for Enterococcus faecalis* which appears to contradict the results of Claesson et al. who found that there is no significant difference between *Enterococcus* and *Staphylococcus* regarding biofilm production [28]. On the other hand, a study that analyzed dual species biofilm, by Ch’ng et al. [32] shows that heme released by *S. aureus* feeds on *E. faecalis* respiration and increases the growth of *E. faecalis* and the overall biomass of the biofilm. In the study, they demonstrate that the heme produced by *S. aureus* is likely to be released in the form of hemoproteins and promotes the formation of *E. faecalis* biofilms, and that *E. faecalis* gelatinase activity facilitates heme extraction from hemoproteins [32]. Based on this information, *Enterococcus* may produce more biofilm than *Staphylococcus*, but more research is needed to confirm this theory.

Regarding the quantity of biofilm produced at different timestamps, in our study *Enterococcus faecalis* produces more biofilm at 6 h-12 h-24 h than 48 h-72 h. Our data contradicts the work of da Silva Fernandes et al. [33] which shows that *Enterococci* at 25 °C and 39 °C produce more biofilm at 48 h than at 24 h. One possible explanation for this is presented by Wamel et al. who found that *Esp* expression (an adhesion molecule) on the surface of the *Enterococcus* bacterial cells varies between strains, depends on growth conditions, and is quantitatively correlated with initial adherence and biofilm formation [34]. Considering the Gram-negative bacteria evaluated in our study, *Pseudomonas* appears to quickly produce biofilms. Liu et al. found that *Pseudomonas* begins to adhere to the contact surfaces and form biofilms early in the first 4 to 6 h and the biofilms begin to be formed in massive amounts after 12 h at 30 °C [35]. Harrison-Balestra et al. showed that the exopolysaccharide of the developing biofilm is visible within 5 h of inoculation and presents the characteristics of a mature biofilm within 10 h [36]. In our study, *Klebsiella pneumoniae* produced biofilm constantly over time, without a specific peak. This finding is supported by the work of Desai et al. who found that there was no difference in the growth rate for producers of strong and weak biofilms; therefore, this means that usually *Klebsiella* produces biofilms in a constant manner over time [37].

Comparing the two Gram-negative bacteria evaluated in our study, *Pseudomonas aeruginosa* seems to produce more biofilm than *Klebsiella pneumoniae*. However, existing data are contradictory over this issue. Stewart et al. found that the biofilm growth rate of *Klebsiella pneumoniae* is twice that of *Pseudomonas aeruginosa* [38]. On the other hand, Moteeb et al. demonstrated using more methods that *Pseudomonas aeruginosa* was able to produce more biofilm than *Klebsiella pneumonia*, which supports our findings [39]. A very important issue regarding this heterogeneity of data is presented by Mahato et al., who showed the importance of the technique used. Their article shows that the biofilm-forming nature on Congo Red Agar is much greater in *Klebsiella pneumoniae* than *Pseudomonas aeruginosa* [40].

In evaluating the biofilm growth rates and total quantity it is important to consider also the intermittent nutrients provided by the dynamic cultivation described in the study and also other requirements considering bacterial metabolism—*Pseudomonas aeruginosa* being an aerobic bacterium while *Enterococcus faecalis* is a facultative aerobic bacterium.

Evaluating the costs for such a device, we considered the raw material and the energy needed to print such a device. One 3D-printed device weighs around 5 g so from one spool of PLA one would be able to print roughly 200 devices. With the average cost of a spool of PLA being around 22.5 euros, the cost of one device is around 0.1125 euros. One device is printed in approximately 40 min and the printer uses about 0.1 kWh. Considering an average of 0.16 euros for 1 kWh, the production cost for one device is 0.016 euros. A combination of both costs suggests that in the end the price for one device is around 0.1285 euros, making it one of the most inexpensive alternatives for the study of BBF in a dynamic way. In addition, being made of PLA, it is biodegradable and thus a perfect alternative for the future.

## 5. Conclusions

In vivo bacterial biofilm formation is an important cause of mortality and morbidity, especially in the hospital environment. It is known that biofilms are involved in many types of infections, usually involving some external medical device being introduced in the human body such as urinary catheters, central venous catheters, orthopedic prostheses, cardiac prostheses and many others. Although great progress has been made in the study of BBF, there are still some unresolved issues, mainly revolving around the lack of standardization, the prohibitive price of materials needed for these studies and the difficulties in interpretation. This paper offers a relatively inexpensive and easy to assemble method for the study of BBF in both static and dynamic ways. Using OCT, FTIR and SEM, we proved that bacterial biofilm can be successfully cultivated with the protocol presented. Being able to offer quality information from such a wide variety of techniques, our device gives valuable versatility for any type of research setting. In addition, having the ability to quantify the bacterial biofilm using one of the most basic methods, such as crystal violet staining, gives the user more data to interpret and analyze, proving its utility. None the less, with an average price for one device being less than 0.15 euros as of 2022, our alternative has the potential to become a standard practice in the future of BBF study.

## Figures and Tables

**Table 1:** The static (3D-printed plates) and dynamic (3D-printed microfluidic devices) measurements of the bacterial biofilm produced by the wild strains (5 of each species) as well as the statistical comparison between the two techniques.

		6 h	12 h	24 h	72 h	120 h	168 h
*Staphylococcus aureus*	Static (mol/L)	7.81	24.82	34.25	46.89	59.31	49.88
	Dynamic (mol/L)	5.05	16.09	28.96	34.94	38.62	33.33
	Mean difference (mol/L)	2.75	8.73	5.28	11.95	20.68	16.55
	Standard error difference	1.14	1.64	3.38	3.48	4.22	2.71
	*p* (*t*-test)	0.043	0.001	0.157	0.009	0.001	<0.001
*Klebsiella pneumoniae*	Static (mol/L)	23.22	27.13	26.44	28.28	23.22	19.54
	Dynamic (mol/L)	13.79	17.47	21.38	21.61	18.39	14.48
	Mean difference (mol/L)	9.43	9.66	5.06	6.67	4.83	5.06
	Standard error difference	1.23	1.67	2.51	1.78	2.63	3.18
	*p* (*t*-test)	<0.001	<0.001	0.078	0.006	0.104	0.151
*Pseudomonas aeruginosa*	Static (mol/L)	24.37	34.48	40.92	78.16	65.06	59.54
	Dynamic (mol/L)	14.94	22.07	29.89	34.94	34.71	38.16
	Mean difference (mol/L)	9.43	12.41	11.03	43.22	30.34	21.38
	Standard error difference	2.16	1.64	3.74	4.88	4.06	4.48
	*p* (*t*-test)	0.002	<0.001	0.018	<0.001	<0.001	0.001
*Enterococcus faecalis*	Static (mol/L)	29.43	41.15	60.00	68.51	56.32	53.56
	Dynamic (mol/L)	6.90	10.11	20.69	26.44	25.98	23.22
	Mean difference (mol/L)	22.53	31.03	39.31	42.07	30.34	30.34
	Standard error difference	1.87	2.88	3.46	3.20	2.76	2.06
	*p* (*t*-test)	<0.001	<0.001	<0.001	<0.001	<0.001	<0.001

## Data Availability

Not applicable.
